# Improving basic skills in celiac-like disease diagnosis: a case report

**DOI:** 10.1186/s12876-018-0894-8

**Published:** 2018-11-03

**Authors:** Vito Domenico Corleto, Vincenza Patrizia Di Marino, Gloria Galli, Giulio Antonelli, Chiara Coluccio, Arcangelo Di Cerbo, Stefania Uccini, Bruno Annibale

**Affiliations:** 1 Gastroenterology and Gastrointestinal Endoscopy Unit, Sant’Andrea University Hospital, “Sapienza” University of Rome, Rome, Italy; 2 Paedriatic Allergology, Allergology Unit, “Policlinico Umberto I” University Hospital, “Sapienza” University of Rome, Rome, Italy; 3 Pathology Unit, Azienda Ospedaliera Sant’Andrea, “Sapienza” University of Rome, Rome, Italy; 4 Digestive Endoscopy Unit, Sant’Andrea University Hospital, “Sapienza” University of Rome, Via di Grottarossa 1035-1039, 00189 Rome, Italy

**Keywords:** Celiac disease; Giardia, Differential diagnosis, Villous atrophy

## Abstract

**Background:**

The diagnosis of Coeliac disease (CD) requires a combination of sign/symptoms, positivity of specific antibodies and duodenal histological evidence of villous atrophy. Duodenal villous atrophy, despite representing the CD landmark, is not specific since it is found in many gastrointestinal disorders. Giardiasis is one of the most common human intestinal protozoan infestations in industrialized countries whose histological duodenal mucosa damage could mimic that of CD. The present report shows how a wise clinical and laboratory assessment led us shortly to a correct diagnosis.

**Case presentation:**

A 42-year-old outpatient woman without previous significant gastrointestinal diseases, was referred with dyspeptic symptoms, fatigue and mild diarrhea from 4 months. Her first investigations including immunoglobulin A (IgA) anti-tissue transglutaminase antibodies (anti-tTG) and stool parasitological and cultural analysis were negative. An esophagogastroduodenoscopy (EGDS) showed no mucosal alteration. But histology demonstrated a Helicobacter Pylori (HP) pan-gastritis while duodenal mucosa showed villous atrophy consistent with a diagnosis of CD Marsh type 3b. While on gluten-free diet (GFD) the patient didn’t experience any improvement of symptoms. Duodenal biopsies were then reviewed showing the presence of trophozoites of Giardia on the luminal surface of the duodenal wall and at the same time, a second stool examination revealed the presence of trophozoites and cysts of Giardia. Treated with metronidazole, 500 mg twice daily for 6 days the patient reduced diarrhea after few days. After about 2 months of GFD she was invited to discontinue it. At the same time stool examination was repeated with negative results. She subsequently performed eradication for Hp with triple therapy (Pylera®). Around 6 months later, the patient did not complain any gastrointestinal symptoms. Serological tests were normal and at a follow-up EGDS, duodenal mucosa had normal histology with normal finger-like villi and absence of Giardia trophozoites.

**Conclusion:**

This case report shows how CD diagnosis can sometimes be manifold. Intestinal villous atrophy alone may not automatically establish a diagnosis of CD. In the present case the clinical scenario could be fully explained by giardiasis. Indeed, different diagnostic tools and a multi-step approaches have been used to determine the final correct diagnosis.

## Background

Coeliac disease (CD) is an immune-mediated enteropathy caused by an immune response to dietary gluten in genetically predisposed individuals. In Europe the estimated median prevalence in the general population is about 1% [1].

The definite diagnosis of CD requires a combination of sign/symptoms, positivity of specific antibodies (anti-transglutaminase and anti-endomysium) and duodenal histological evidence of villous atrophy associated to crypt hyperplasia and intraepithelial lymphocytosis [2].

Due to its heterogeneous manifestations, the relatively high percentage of false negative results of specific antibodies [3] and histological evaluation, CD diagnosis is sometimes challenging. In addition, the presence of duodenal villous atrophy, despite representing the CD landmark, is not specific since it could be a sign of other gastrointestinal disorders [4].

For these reasons, further investigations such as genetic evaluation (HLA) and/or gluten challenge with subsequent follow-up esophagogastroduodenoscopy with duodenal biopsies including the research of parasitic infections can be useful in selected cases.

Concerning our case, it is known that giardiasis is one of the most common human protozoan infestations in industrialized countries [5] with selective intestinal tropism, particularly for the duodenum, where the histological damage could mimic that of CD.

We aim to illustrate how a strict clinical observation led us to clarify in a short time a misdiagnosis of CD.

## Case presentation

A 42-year-old woman, married and with two children, was referred to our hospital outpatient gastrointestinal clinic for a 4 months’ history of post-prandial heartburn with frequent regurgitations, fatigue and change in bowel movements (4–5 soft stools). At the moment of the first consult, a written informed consent on the publication of personal information was obtained from the patient. She had experienced unintentional weight loss of 3 kg in about one month despite normal or even increased food intake. In her clinical history no previous significant gastrointestinal symptoms were present. The patient’s older sister had been diagnosed of coeliac disease at age 20. Her personal and family history was otherwise unremarkable. She first underwent biochemical investigations including immunoglobulin A (IgA) anti-tissue transglutaminase antibodies (anti-tTG) and stool parasitological and cultural analysis. Serological testing showed normal IgA levels and negativity for anti-tTG levels and antiendomysial antibodies (Ema). The patient’s ferritin was 33 mg/l (n.v. 30–400 mg/l), serum folate was lower than 5 nmol/l (n.v. > 7 nmol/l), haemoglobin level was normal as well as white blood cells and platelet count. The result of stool analysis was negative for parasites and ova. Since symptoms persisted, she was then referred for an upper GI endoscopy. Esophageal and gastric mucosa did not show any macroscopic alterations. Duodenal folds were normally represented as well as mucosa. Multiple gastric antrum and corpus-fundus mucosa biopsies were taken along with biopsies from the bulb and second part of duodenum (at least four).

**Fig. 1:**
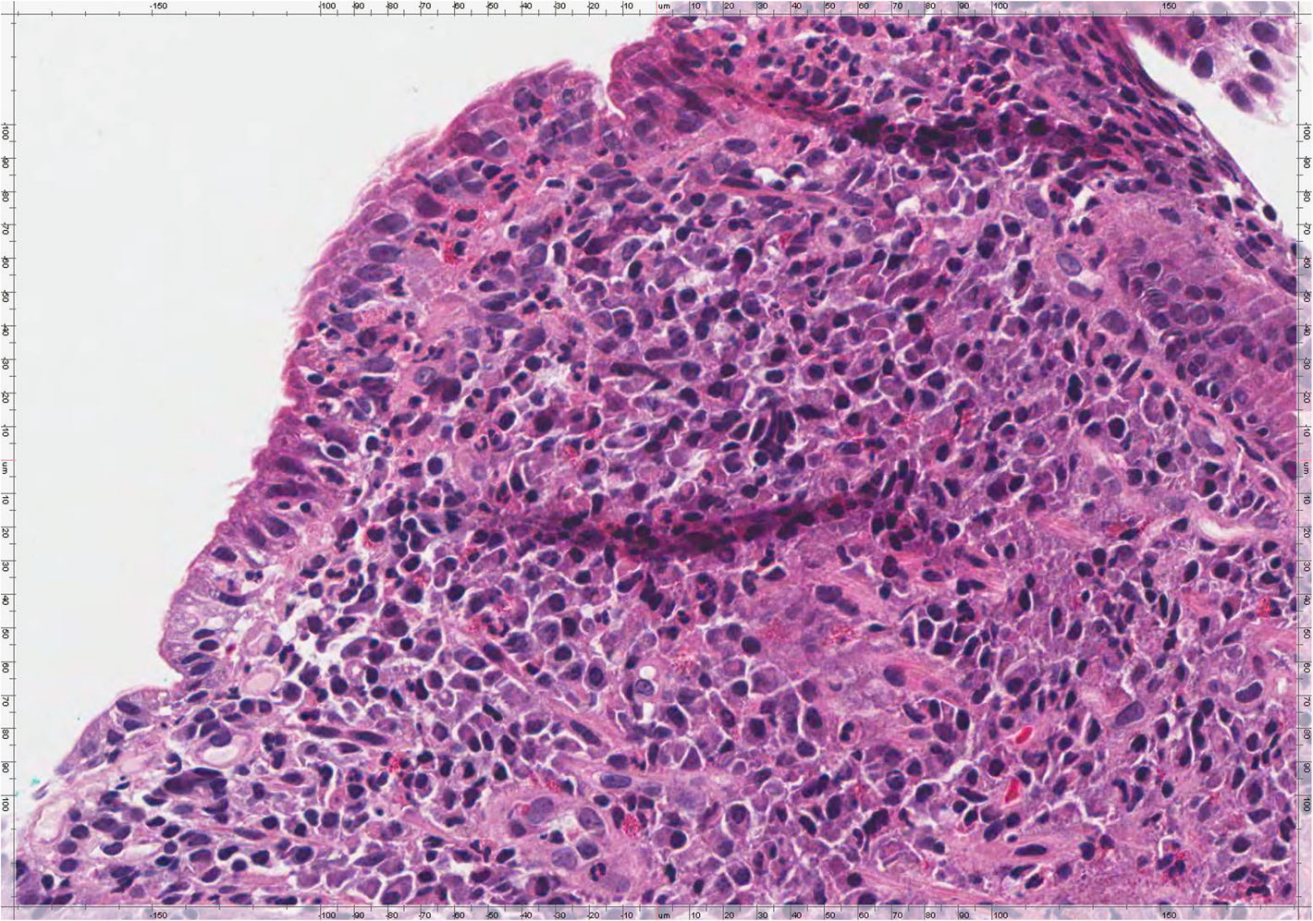
Duodenal mucosa showing villous atrophy associated with increased intraepithelial lymphocyte (IEL) numbers and an unusual large number of intraepithelial granulocytes. The lamina propria is completely filled by chronic inflammatory cells with some scattered eosinophils (H&E, × 400)

**Fig. 2:**
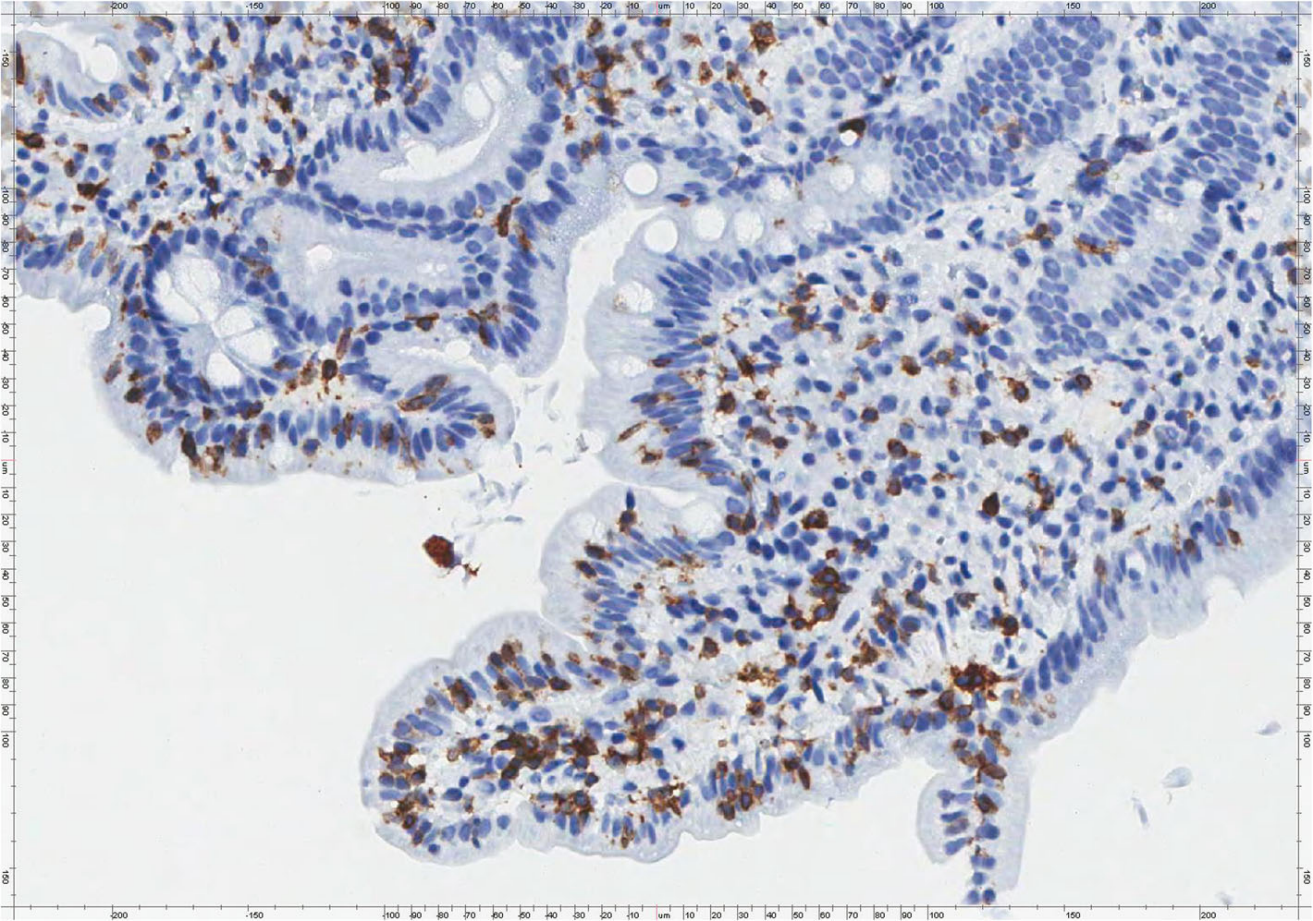
CD3 immunostaining of duodenal mucosa showing an increased number of CD3+ T IEL (× 320)

**Fig. 3:**
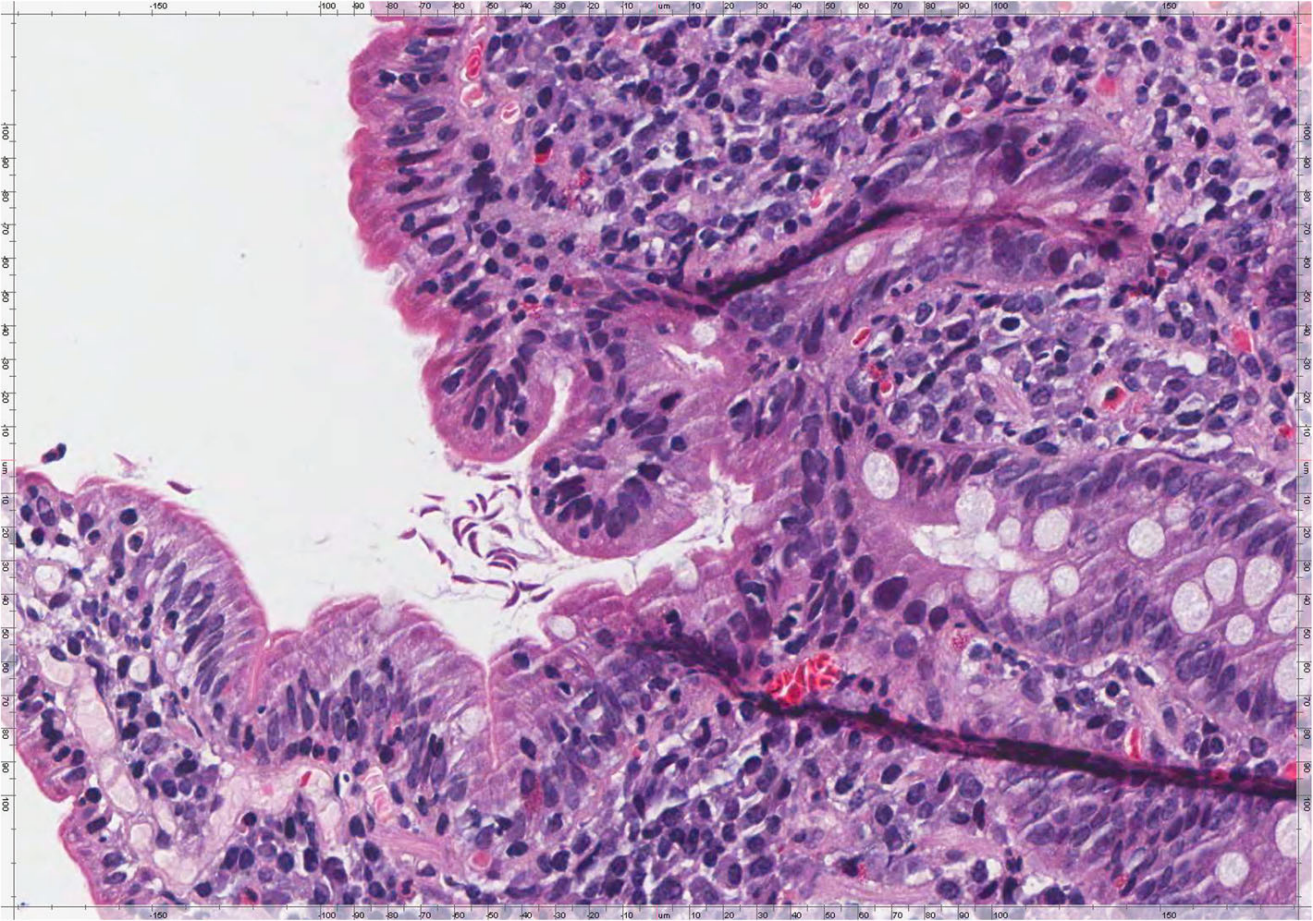
Duodenal mucosa showing the presence of scattered crescent-shaped randomly oriented trophozoites of Giardia on the luminal surface of the duodenal wall (H&E, × 400)

Gastric biopsies showed a Helicobacter Pylori (HP) pan-gastritis while duodenal mucosa showed villous atrophy (Fig. 1) associated with an increase in intraepithelial T lymphocyte (IEL) numbers up to more than 40 IEL/100 epithelial cells (EC), recognized by CD3 immunostaining (Fig. 2). The histologic features were consistent with a diagnosis of coeliac disease Marsh type 3b [6, 7]. She was informed of the result and advised to begin gluten-free diet (GFD). After 4 weeks of GFD the patient didn’t experience any improvement of symptoms, and bowel movements with abdominal pain increased to around 6–7 daily; she was advised to prompting repeat further stool examinations. Genetic evaluation for alleles HLA specific for coeliac disease was also requested. At the same time, in order to re-evaluate initial diagnosis, duodenal biopsies were reviewed and a careful study of the duodenal mucosa showed the presence of scattered crescent-shaped randomly oriented trophozoites of Giardia on the luminal surface of the duodenal wall (Fig. 3). The organisms were minute, easily overlooked or mistaken for detached intestinal epithelial cells or erythrocytes. Subsequently, results of the second stool examinations revealed the presence of *G. lamblia* with findings of trophozoites and cysts. The patient was consequently treated with metronidazole, 500 mg twice daily for 6 days, showing a prompt response with a reduced frequency of diarrhea in the following days. The genetic results showed DQA1*03 and DQB1*03:02 alleles codifying for HLA-DQ8, otherwise compatible with CD diagnosis*.* In the following weeks, the patient intermittently maintained a GFD, but observed no difference in her well-being. Stool examination was repeated 2 months after the end of antibiotic therapy with negative results. After about 2 months of GFD she was invited to discontinue it. She subsequently performed eradication therapy for HP with triple therapy of metronidazole, tetracycline and bismuth (Pylera®).

**Fig. 4:**
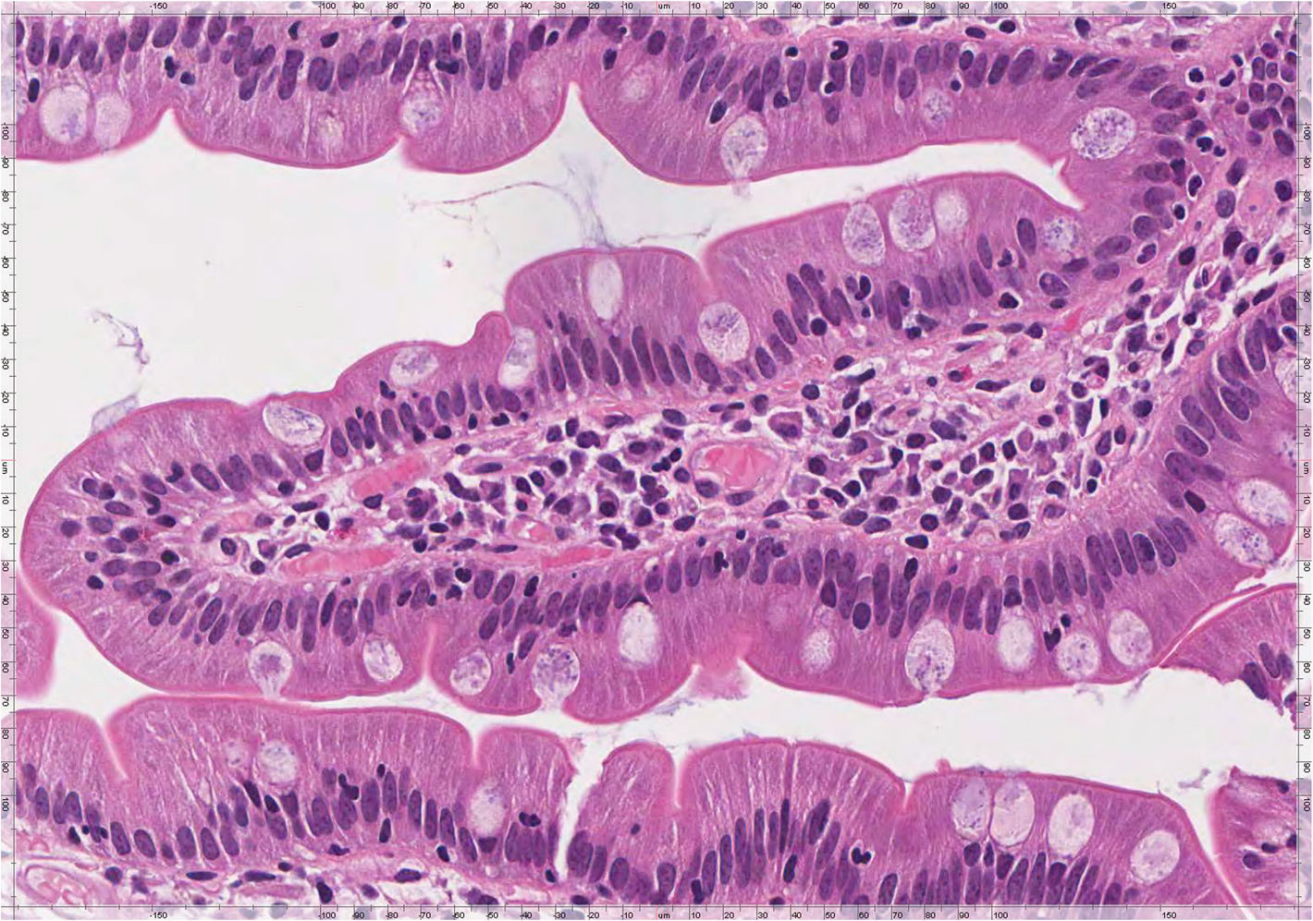
Duodenal mucosa showing a normal histologic appearance with normal finger-like villi, no evidence of increased IEL numbers and complete absence of crescent-shaped Giardia trophozoites (H&E, × 400)

Around 6 months later, the patient did not complain gastrointestinal symptoms. In January 2017 she repeated serological tests and a follow-up esophagogastroduodenoscopy. No alterations were found in haemoglobin, folic acid, cholesterol, triglycerides and antibodies (anti-tTG and Ema) levels. Multiple duodenal biopsies were performed, showing normal histologic appearance of the mucosa with normal finger-like villi, no evidence of increased IEL numbers and complete absence of crescent-shaped Giardia trophozoites (Fig. 4). The gastric biopsies showed resolution of active HP gastritis. The patient showed no clinical signs of CD and the conclusion was that the clinical scenario could be fully explained by giardiasis.

## Discussion and conclusions

This case report demonstrates how differential diagnosis can be challenging in coeliac disease diagnostic work-up. Differently from the past, presence of duodenal villous atrophy cannot be defined as coeliac disease landmark at first sight [4]. Nowadays, the morphologic/histological changes in coeliac disease are characteristic but not specific and they must be evaluated in conjunction with clinical and laboratory evidence such as malabsorption, specific serum antibody levels and response to a gluten-free diet [8]. Various other pathological conditions mimicking coeliac disease may cause malabsorption syndrome [9]. In Giardiasis, the villous architecture is usually normal (96% of investigated patients) [5]*,* mainly affecting the lamina propria, in which lymphoid hyperplasia and increased numbers of chronic inflammatory cells and eosinophils are seen. Sometimes, *Giardia lamblia* can induce increase of duodenal intraepithelial lymphocytes associated or not to crypt hyperplasia and different grades of villous atrophy [10–12]. These alterations lead to a significant range of symptoms going from abdominal chronic pain to diarrhea and signs of malabsorption [13].

In this case, the mucosa was mainly affected by evident villous atrophy associated with massive intraepithelial lymphocytosis and rich infiltration in the lamina propria by inflammatory cells. On this basis, the histologic features were more consistent with a diagnosis of coeliac disease rather than of duodenal Giardia infection.

However, only accurate clinical information associated with a thorough evaluation of duodenal mucosa was able to avoid misdiagnosis of coeliac disease in the present case of *Giardia lamblia* infection.

Discerning coeliac disease from Giardiasis is pivotal in order to proceed to adequate treatment. In this specific case report, strict follow-up played a key-role in achieving the final diagnosis in a limited time. In the present case, re-evaluation of clinical history and the further investigations carried out allowed to reach a final correct diagnosis in a relatively short period of time.

Recent case series and systematic reviews have shown that a wrong CD diagnosis can be reached in a consistent percentage of seronegative patients [3, 12]. In fact, a recent study on 200 seronegative patients showed a percentage of seronegative non-coeliac disease patients of 69% [14]. These patients had different causes of villous atrophy, such as giardiasis, drugs mucosal damage, Crohn disease and autoimmune enteropathy; even if some cases remained unknown [12, 14]. Until 10 years ago, prevalence of seronegative CD was reported to be around 10 to 20% [15, 16]. Nowadays, due to the increased sensitivity of serological assays (anti-tTG and Ema detection) and to the acknowledgement of different alternative diagnosis of villous atrophy and their specific diagnostic work-up, this percentage decreased to 3–5% of the total number of patients with a real diagnosis of CD [12, 17].

For these reasons, clinical, biochemical and histological re-evaluation is mandatory in some settings. Also γδ intraepithelial lymphocytes evaluation through flow cytometry could be a useful tool to exclude differential diagnosis [18].

It has been reported that about 0.11% of patients of industrialized countries presented with overt Giardia duodenalis infection [5]. However, the same countries have shown a giardia seroprevalence up to 2%. The gold standard for Giardia infection diagnosis is the microscopic analysis of fecal samples in order to search for cysts and trophozoites [19], but false-negative results are commonly found due to variable parasite excretion, cyclicity of cysts or due to a low number of organisms present in the sample [20]. Considering also our case report, in case of high suspicious of parasitic infection, we suggest the examination of 3 stool samples or other diagnostic tests such as ELISA (enzyme-linked immunosorbent assay), rapid tests (immunochromatographic tests), and the detection of *Giardia* specific genes by PCR in order to avoid false negative results.

In conclusion, our case report underlines how villous atrophy alone may not automatically establish a diagnosis of celiac disease. Indeed, since intestinal villous atrophy can be associated to other intestinal and/or systemic diseases, when considering coeliac disease and its differential diagnosis, a pivotal importance must be recognized to clinical scenario and antibodies positivity. This case report clearly shows how CD diagnosis can sometimes be manifold. Indeed, different diagnostic tools and a multi-step approaches have been used to determine the final diagnosis.

## Abbreviations

Anti tTG: Anti-tissue transglutaminase antibodies
EGDS: Esophagogastroduodenoscopy
Ema: Antiendomysial antibodies
GFD: Gluten-free diet
HP: Helicobacter Pylori
IEL: Intraepithelial T lymphocyte
IgA: Immunoglobulin A
